# Dietary outcomes of a community based intervention for mothers of young children: a randomised controlled trial

**DOI:** 10.1186/s12966-014-0120-1

**Published:** 2014-09-23

**Authors:** Jonine Maree Jancey, Sarojini Maria Dos Remedios Monteiro, Satvinder S Dhaliwal, Peter A Howat, Sharyn Burns, Andrew P Hills, Annie S Anderson

**Affiliations:** Western Australian Centre for Health Promotion Research, School of Public Health, Curtin University, Western Australia, Australia; Mater Mothers Hospital and Mater Medical Research Institute- UQ, Queensland, Australia; Centre for Musculoskeletal Research, Griffith Health Institute, Griffith University, Queensland, Australia; Centre for Public Health Nutrition Research Centre for Research into Cancer Prevention and Screening, Division of Cancer Research Medical Research Institute, Level 7, Mailbox 7, University of Dundee Ninewells Hospital and Medical School, Dundee DD1 9SY, Dundee, Scotland, UK

**Keywords:** Community interventions, Behaviours, Mothers, Nutrition

## Abstract

**Background:**

Unhealthy dietary behaviours are one of the key risk factors for many lifestyle-related diseases worldwide. This randomised controlled trial aimed to increase the level of fruit, vegetable and fibre intake and decrease the fat and sugar consumption of mothers with young children (0–5 years) via the playgroup setting.

**Methods:**

Playgroups located in 60 neighbourhoods in Perth, Western Australia were randomly assigned to an intervention (n = 249) or control group (n = 272). Those in the intervention group received a 6-month multi-strategy primarily home-based physical activity and nutrition program (data is only presented on dietary behaviours). Data on dietary consumption was collected via the Fat and Fibre Barometer and frequency of serves of fruit and vegetable and cups of soft drink, flavoured drink and fruit juice. The effects of the intervention on continuous outcome measures were assessed using analysis of variance (ANOVA), after adjusting for mother’s age and the corresponding variables.

**Results:**

The outcomes of the intervention were positive with the intervention group showing statistically significant improvements, when compared to the control group in the overall consumption of fat and fibre (p < 0.0005); of fibre (p < 0.0005) – fruit and vegetables (p < 0.0005), wholegrain (p = 0.002): and fat (p = 0.005) – dairy products (p = 0.006) and lean meat and chicken (p = 0.041). There were no significant changes in the consumption of sweet drinks.

**Conclusions:**

This intervention was successful in improving dietary intake in the intervention group participants. The moderate positive outcomes indicate that playgroups potentially provide quite a viable setting to recruit, engage and retain this hard to reach group of mothers of young children in programs that support the adoption of health-enhancing behaviours. This adds valuable information to this under researched area.

**Trial registration:**

Australian and New Zealand Clinical Trials Registry ACTRN12609000718246

## Background

Unhealthy dietary behaviours are one of the key risk factors for many lifestyle-related diseases worldwide [1–3]. Globally, 1.8% of the disease burden is attributed to inadequate fruit and vegetable consumption [4], while in Australia this figure is estimated to be 2.1% [5]. The economic costs associated with unhealthy dietary behaviours are substantial [6] and as the prevalence of lifestyle-related diseases such as obesity and type II diabetes increase the associated economic costs are predicted to rise significantly [7]. Increasing healthy dietary behaviours is recognised as the single most important aspect of reducing an individual’s risk of lifestyle-related disease [6].

According to the Australian Dietary Guidelines, it is recommended that individuals enjoy a wide variety of nutritious foods, including fruit and vegetables and limit their intake of foods containing saturated fat, added salt, added sugars and alcohol [7]. Guidelines suggest that adult women should consume two serves of fruit, five serves of vegetables, six serves of grains, two and a half serves of dairy foods or alternatives and two and a half serves of lean meat and poultry, fish, eggs or alternatives. During pregnancy and the postpartum period, these recommendations change to reflect an increased need for nutrients, vitamins and minerals.

However, global rates of fruit and vegetable consumption are low [8]. For example, in the United States approximately 74% women of childbearing age (25 to 44 years) reported consuming less than five serves of fruits and vegetables, while in the United Kingdom this figure may be between 73% and 77% [9]. In the 2011–12 Australian Health Survey [10], 55% of Australian women aged 24 to 44 did not meet the recommended intake of two serves of fruit and 92% consumed less than the recommended five serves of vegetables. The most recent Australian national data on dietary fibre obtained from the 1995 National Nutrition Survey indicates that the fibre intake of adult women of 20 g/day is less than the recommended 25 g/day [11].

Furthermore, many Australians are over consuming foods that are high in sugar and/or fat [12], with energy dense, nutrient poor (EDNP) foods, often referred to as ‘extra’ foods (e.g. sugary soft drinks; pies/pastries; wine) contributing to 33.8% of Australian womens’ mean daily energy intake [13]. This over consumption of ‘extra foods’ has contributed to the significant increase in the prevalence of overweight and obesity in Australian women of childbearing age, with 35% of women aged 18 to 24 years being classified as overweight or obese, increasing to 55% of women aged 35 to 44 years [14].

Factors influencing the food choices of mothers with young children are varied. Barriers to consuming a healthy diet include inadequate food related knowledge and preparation skills, [15] affordability and access to healthy produce [16], food choices based on convenience due to reduced time for meal preparation [17] and a greater focus on the family [18]. Conversely, it has been acknowledged that during this period mothers of young children may experience increased motivation to adopt healthier behaviours [19] thereby providing a window of opportunity when women may be more receptive to nutrition messages stemming from health concerns [20].

There are limited dietary interventions aimed at mothers with young children. Interventions that have been implemented have predominantly focused on weight loss outcomes as opposed to dietary behaviours and have targeted women with high body mass index scores [21–24]. The overall aim of this RCT was to improve dietary intake and increase the physical activity levels of mothers’ with young children via a flexible home based multi-strategy intervention. This research paper will specifically report on the outcomesrelated to increasing fruit, vegetables and fibre intake and decreasing the fat and sugar-sweetened beverage consumption.

## Methods

### Design and intervention

The 6-month RCT was informed by a pilot project with regards to recruitment, retention and behaviour change intervention strategies via playgroups [25]. Playgroups provide a community-based venue for mothers’ of childen aged less than five years to meet and socialise in a relaxed and informal environment. The playgroup sessions are run by parents and are usually held once a week for a two-hour period. All playgroups in Western Australia (WA) are registered with Playgroups WA, an incorporated body and are not for profit.

The nutrition content of the intervention was based on the Australian Dietary Guidelines [26] and behaviour change strategies were informed by the Social Cognitive Theory [27], Trans-theoretical model [28] and motivational interviewing [29]. Behaviour change theory techniques included increasing self-efficacy, provision of nutrition information and discussion of solutions to barriers to healthy eating; increasing understanding of strategies to obtain support from family and friends; increased support for behaviour change through encouragement; skill building, rewards, positive self-talk, goal setting, monitoring and relapse prevention strategies (see Table 1). The intervention was primarily home-based and supported by five face-to-face workshops (30-minute sessions every month) at playgroups that provided an opportunity for the resources to be further explained and topics clarified. The face-to-face sessions were conducted by final year Health Science students recruited through local universities and professional associations. The program resources included a comprehensive specifically tailored information booklet, menu planner, nutritional information panel guide, guidelines for the formulation of a shopping list, recipe booklets and bi-monthly ‘chatty’ newsletter providing health information and health related activities. The control group completed a baseline and post-intervention questionnaire and had no other contact. Further information about the physical activity outcomes of this study has been published elsewhere [30], with this paper focussing on the dietary outcomes of the intervention.

**Table 1:** **Description of intervention linked to behaviour change theory**

**Theme**	**Intervention**	**Theory**
Orientation to the diet intervention (Week 1)	Distribution of resources (booklet, menu planner, recipe booklet) containing information on healthy eating (increasing fruit, vegetables and fibre and reducing fat and sugar-sweetened beverages)	expectation and expectancies; Self-efficacy (SCT)
	Barriers and benefits to a healthy diet and overcoming barriers	
Behaviour change (week 5)	Goal setting - diet	behavioural capabilities; self-efficacy
	Family dinner planner & food record sheet	Observational (SCT and TTM)
	Activity with healthy dinner planner	
	Newsletter	
Monitoring progress (week 9)	Review established goals	behavioural capabilities; self-control; social support; reciprocal determinism; reinforcement (SCT); MI
	Set new short term goals	
	Support networks	
	Review resources	
	Newsletter	
Monitoring progress (week 13)	Review established goals	self-control; social support; reciprocal determinism(SCT); MI
	Set new short term goals	
	Menu planning	
	Shopping list with healthy tips	
	Reading food labels	
	Newsletter	
Reinforcing messages/information (week 17)	Overcoming relapses	Social support; observational, reinforcement (SCT)
	Support networks	
	Modify recipes to make healthier	
	Healthy cooking methods	
	Newsletter	
Review and feedback (week 21)	Review of goals; review of program;	Social support; observational, behavioural capabilities (SCT)
	Fibre and glycaemic index	
	Modified recipes/healthy cooking methods	
	Newsletter	

### Recruitment and randomization

**Figure 1:**
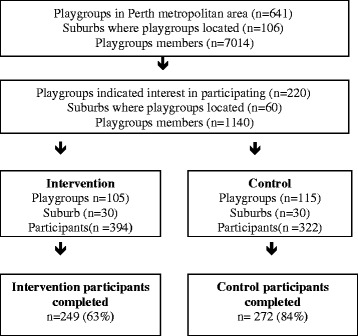
**Flow chart of recruitment process.**

Playgroups located in 60 suburbs (neighbourhoods) in the Perth Metropolitan area in Western Australia (WA) registered with Playgroups WA were randomly assigned to the intervention (n = 30) or control (n = 30) group and arbitrarily matched on their Socio-Economic Indexes For Area (SEIFA) scores [14], a value derived from income, education level, employment status, and skill level. Playgroup WA staff contacted the playgroups to obtain consent for the project staff to make contact. To be eligible for participation in the study, participants were required to be: women aged 18 years and above; have at least one child aged 0–5 years; and on no special diet. Of the 1140 participants who were recruited, 716 participants consented to be part of the study (see Figure 1).

Ethics approval was obtained from the Curtin University Human Research Ethics Committee (approval number HR 183/2008). Trial Registration: Australian and New Zealand Clinical Trials Registry ACTRN12609000718246.

### Nutrition measurements

Dietary intake of participants was collected by the validated Fat and Fibre Barometer (FFB) [31]. The FFB is a brief food behaviour questionnaire that is self-administered and contains 20 food related behaviour items. It has good internal consistency (α = 0.86) and test retest reliability (r = 0.92). The relative validity of the FFB was assessed by comparing it to the food frequency questionnaire with weighted Kappa indicating fair to moderate agreement. The FFB assesses individual’s fat-related food intake (fried foods, dairy foods, meat and chicken and butter) and fibre-related food intake (wholegrain foods, fruit and vegetables). Response values for each item range from 1 to 5, with ‘1’ representing food behaviour associated with the high fat intake or low fibre intake, to ‘5’ representing the low fat or high fibre intake. Fat and fibre scores are calculated by summing the scores from the corresponding fat and fibre foods assessed. Individual items (fruit and vegetables; wholegrain foods; dairy products; lean meat and chicken) were also analysed.

Additional self-administered questions assessed the frequency of serves of fruit and vegetable intake per day and cups of soft drink, flavoured drink and [32], fruit juice consumed per day [33]. Fruit and vegetable serves were defined in the questionnaire. One serve of vegetables is equivalent to ‘1 cup (75 g) of cooked vegetables or legumes, 1 cup of salad vegetables, 1 small potato’ and one serve of fruit is equivalent to ‘1 medium piece (150 g) of fruit, 1 cup of diced pieces or canned fruit, cup of fruit juice [26]. Demographic data was also collected.

### Statistical analysis

Descriptive statistics are reported as the mean (±SD) for continuous data and count and percentages for categorical data (Table 1). The effects of the intervention on continuous outcome measures were assessed using analysis of variance (ANOVA), after adjusting for mother’s age and the corresponding variable at baseline (Table 2). Also, playgroups effects were treated as “block” random effects within the analysis of variance and the variability between these blocks were removed before valid comparisons between the two treatment groups were made to remove the effect of clustering by playgroups [34]. Figure 1 shows the percentage difference between the intervention and control group for Fat and Fibre Barometer and consumption of food types. Statistical analyses were performed using Statistical Package for Social Sciences (SPSS Version 20).

**Table 2:** **Baseline characteristics of intervention and control groups**

**Variables**	**Intervention**	**Control**	**P-value**
	**N = 249**	**N = 272**	
AGE	35.9 ± 4.3	35.6 ± 4.3	n.s.
BMI (kg/m^2^)			0.081
<25	73 (54.5%)	116 (67.1%)	
≥25 and <30	47 (35.1%)	44 (25.4%)	
≥30	14 (10.4%)	13 (7.5%)	
Vegetable consumption			n.s.
Below recommendation	212 (85.1%)	215 (79.0%)	
Met Recommendation	35 (14.1%)	55 (20.2%)	
Above Recommendation	2 (0.8%)	2 (0.7%)	
Fruit consumption			0.036
Below recommendation	157 (63.3%)	143 (52.6%)	
Met Recommendation	75 (30.2%)	101 (37.1%)	
Above Recommendation	16 (6.5%)	28 (10.3%)	
Pregnant, breastfeeding or postpartum	103 (41.4%)	95 (34.9%)	n.s.
Parity			n.s.
1 child	82 (32.9%)	94 (34.6%)	
≥2 child	167 (67.1%)	178 (65.4%)	
Married or De facto	245 (98.8%)	265 (98.1%)	n.s.
University degree or higher	127 (51.0%)	170 (62.5%)	0.008
Not employed	99 (39.8%)	94 (34.7%)	n.s.
Annual household income (AUD)			n.s.
<$51,000	32 (13.4%)	27 (10.1%)	
≥$51,000 to < $101,000	95 (39.9%)	107 (39.9%)	
≥$101,000	111 (46.6%)	134 (50.0%)	
SEIFA score			<0.0005
Least disadvantaged	95 (38.6%)	147 (54.9%)	
Less disadvantage	83 (33.7%)	21 (7.8%)	
Average disadvantage	34 (13.8%)	55 (20.5%)	
Disadvantaged	20 (8.1%)	36 (13.4%)	
Most disadvantaged	14 (5.7%)	9 (3.4%)	

Abbreviations: *BMI* body mass index, *SEIFA* socio-economic index for area. n.s.: p-value > 0.05.

Adjusted for baseline value of the corresponding variable and mothers age.

## Results

In total, 521 participants completed both the baseline and post-program questionnaire (72.8% retained). The intervention group compared to the control group had slightly higher BMI, lower fruit consumption ‘above recommendation’ , and had a lower percentage of participants who were ‘least disadvantaged’ (SEIFA score). These differences, if of any effect, bias the results against the Intervention group and hence do not compromise the validity of the research. No significant differences at baseline were present for all other variables (p > 0.1) (see Table 2).

The continuous diet outcome variables and Fat and Fibre Barometer variables were compared between the intervention and control group post intervention (see Table 3). The intervention group was significantly higher than the control group on the Fat and Fibre Barometer, Fibre Barometer, fruit and vegetables, whole grain foods, Fat Barometer, dairy products, and meat and chicken (all *p* < 0.05). These mean difference between the intervention and control ranged from 0.12 to 0.17. Intervention group participants also consumed a higher number of serves of fruit and vegetables compared to the control group. The mean differences between the intervention and control ranged from 0.16 to 0.35. No significant differences were reported between the two groups in the consumption of fruit juice drinks, soft drinks and flavoured drinks.

**Table 3:** **Comparison diet outcomes between intervention and control groups**

**Scores**	**Intervention**	**Control**	**Mean difference**	**95% CI of mean difference**	**P-value**
**Fat and fibre barometer**					
Fat and fibre barometer	3.63 ± 0.02	3.52 ± 0.02	0.12	0.07, 0.16	<0.0005
Fibre barometer	3.47 ± 0.03	3.29 ± 0.02	0.17	0.10, 0.24	<0.0005
Fruits and vegetables	3.39 ± 0.03	3.23 ± 0.03	0.16	0.08, 0.24	<0.0005
Wholegrain foods	3.55 ± 0.04	3.39 ± 0.04	0.16	0.06, 0.26	0.002
Fat barometer	3.73 ± 0.02	3.65 ± 0.02	0.08	0.03, 0.14	0.005
Dairy products	3.37 ± 0.04	3.21 ± 0.04	0.17	0.05, 0.29	0.006
Lean meat and chicken	3.93 ± 0.04	3.81 ± 0.04	0.12	0, 0.24	0.041
**Consumption per day**					
Fruits (serves)	2.26 ± 0.05	2.10 ± 0.05	0.16	0.01, 0.31	0.038
Vegetables (serves)	3.39 ± 0.08	3.05 ± 0.08	0.35	0.13, 0.56	0.002
100% fruit juice (serves)	0.18 ± 0.04	0.13 ± 0.03	0.06	−0.04, 0.16	0.239
Soft drinks (cups)	0.18 ± 0.05	0.25 ± 0.05	−0.07	−0.20, 0.06	0.309
Flavoured drinks (cups)	0.18 ± 0.03	0.21 ± 0.02	−0.02	−0.09, 0.05	0.517

Comparison between groups after adjustment for baseline value of the corresponding variable and mother’s age.

**Figure 2:**
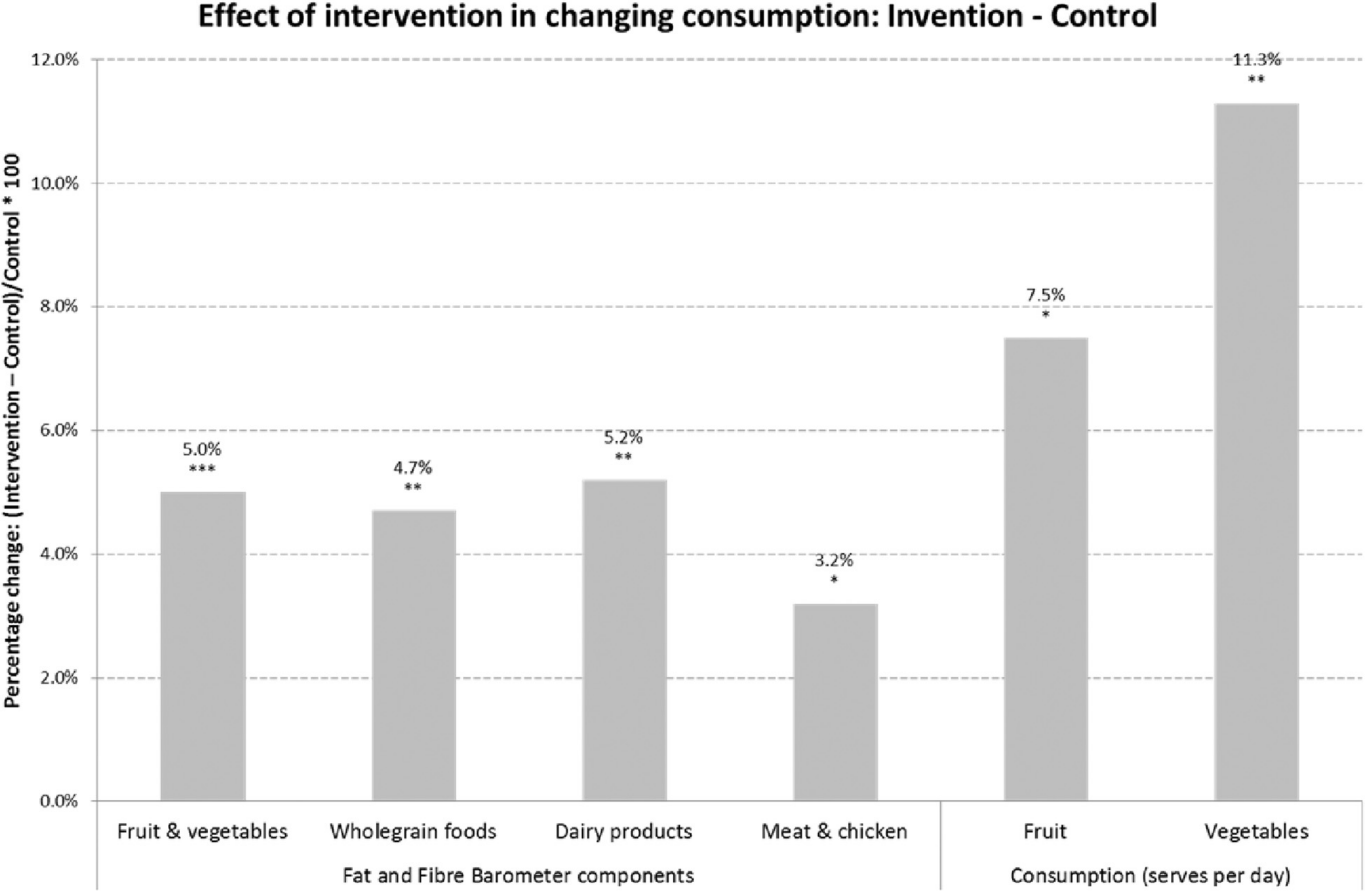
**Effect of intervention in changing consumption: Intervention -Control.**

The effect of the intervention as compared to the control group in changing consumption is summarised in Figure 2, illustrating that the intervention increased positive behaviours in consumption by between 3.2% and 11.3% (increased consumption of lean meat and chicken (by 3.2%); wholegrain foods (by 4.7%); fruit and vegetables (by 5.0%) and decreased consumption in dairy product (by 5.2%)). Daily serve of fruit (by 7.5%) and vegetables (by 11.3%) also increased.

## Discussion

This study was conducted via a playgroup setting, in order to reach mothers of young children to provide them with a flexible home-based multi-strategy intervention. The intervention aimed to encourage an increase in their levels of fruit, vegetable and fibre intake and a decrease in their fat and sugar-sweetened beverage consumption. The outcomes of the intervention were moderately positive suggesting the program to be both acceptable and suitable for the mothers of young children.

Overall, the Intervention group improved their consumption of Fat and Fibre (*p* < 0.0005) along with their Fibre (*p* < 0.0005) and Fat (*p* = 0.005) consumption. The reported increase in the Intervention groups’ consumption of fruit and vegetables (*p* < 0.0005) and wholegrain (*p* = 0.002) were encouraging considering the low levels of fruit and vegetable consumption worldwide (e.g. Australia, US, UK) [10,11,15], as well as fibre [11]. However, it should be acknowledged that the actual changes in daily consumption were small and although the participants did achieve the recommended daily serves of fruit at the conclusion of the intervention, they did not achieve the recommended intake for vegetables, with the reported mean intake being 3.39 serves for day. This dietary area requires more focus and additional investigation to determine ways of increasing daily vegetable intake.

The statistical significant decrease in Fat *(p* = 0.005) that included dairy products (*p* = 0.006) and lean meat and chicken (*p* = 0.041) was encouraging as it is well recognised that the intake of fat is high and above recommended levels [12]. However, in regards to sugar-sweetened drinks (soft drinks, fruit juices and flavoured drinks) there were no significant changes found between groups. This result is not unexpected as the intervention placed little emphasis on this aspect of diet compared to fruit and vegetable intake, and fat reduction. Nevertheless, this area warrants further investigation as sugar-sweetened drinks such as soft drinks and juices are a common source of excess sugar, contributing to weight gain and tooth caries [35].

However, the moderate positive outcomes in regard to fat and fibre intake indicate that playgroups potentially provide quite a viable setting to recruit, engage and retain this hard to reach groups of mothers of young children in programs that support the adoption of health-enhancing behaviours [25]. More aggressive recruitment strategies, such as more personalised contact may serve to improve these outcomes. It also indicates the suitability of an intervention program that is flexible and primarily home-based, but also incorporates supportive face-to-face information and skill building sessions in a relaxed, family friendly group environment. This intervention was conducted in a ‘real world context’ using a combination of strategies, which strengthened the program’s appeal and ability to influence.

The outcomes are encouraging when the many barriers to maintaining a healthy diet that confront women of young children are considered. These include the changing priorities and competing family demands [18] reduced time for meal preparation [22] affordability and access to healthy foods [16], and at times a lack of food related knowledge and preparation skills [16]. This study comprised a range of supportive strategies able to help women make positive changes to their diet over a 6-month period. These findings provide further support for the notion that this period of early motherhood provides a ‘window of opportunity’ for encouraging the adoption of healthier behaviours [19], with the potential for this to have a beneficial impact on offspring.

These research outcomes are not dissimilar to the Women’s Diabetes Reduction Study [36] and the Women Infants and Children Study [37]. Both studies also showed statistically significant results at six and eight months post baseline, respectively. However, as with the findings of this research, the changes in daily consumption by the intervention group of vegetables, fruit and fibre were small. Thompson et al. [36] reported an increased vegetable consumption of only 0.31, while Havas and colleagues [37] combined fruit and vegetables serving daily intakes increased by 0.10. These interventions [36,37] both targeted women, adopting similar behavioural models and strategies as used in this intervention. The theories included the Social Cognitive Theory and the Transtheoretical Model and they developed strategies that encouraged support, reduced barriers, providing knowledge and skills, incorporating goal setting and monitoring. The strategies supporting these theories also included face-to-face interventions, along with supportive information resources e.g. newsletter and written resources. Both studies acknowledged the importance of flexibility and a multi-strategy approach as a key component of these programs.

To the best of our knowledge, this nutrition behaviour change intervention may be the largest RCT to have specifically targeted mothers with young children aged 0 to 5 years [23,38,39]. A very limited number of intervention studies have been reported where all the participants were mothers with at least one young child [24,37–43], compared to those interventions that included only a small proportion of mothers with young children [36,37,44–50]. This makes this study very timely, relevant and a welcome contribution to the dietary intervention literature, providing a practical workable model to inform others.

### Limitations

Self-report surveys were used to obtain data on the consumption of fruit, vegetable, fat and fibre and sugar (via consumption of sugar-sweetened beverages), which may have led to some over-reporting. However, this potential bias was minimised by the use of a control group that would have responded in a similar way. The combining of both the physical activity and nutrition components into this intervention may have diluted the outcomes and it may have been better to focus on one behaviour (nutrition outcomes are only reported here). There was no endpoint to the study in the form of measurement of changes in weight, however, we chose not to focus on this, instead focussing on the positive aspects of a eating a healthy diet. Also this study’s measurement of change in behaviour is limited to a 6-month timeframe.

## Conclusions

This intervention was successful in recruiting women into a 6-month flexible and predominantly home-based nutrition intervention. It was effective in achieving its aim of increasing fibre and decreasing fat in the intervention group participants, however, it did not influence sugary drink consumption and the recommended daily serves of vegetables was not achieved. However, in this instance it was found that playgroups provide a sound avenue for reaching and recruiting women into health programs and in turn equipping them with skills and information. This intervention adds to the research in terms of the paucity of effective interventions for mothers with young children and indicates the usefulness of playgroups as a vehicle for future programs. Further research is required in this area.

## Abbreviations

BMI: Body mass index
SEIFA: Socio-economic index for area
NS: Non-significant

## References

[1] World Health Organization (2008). 2008–2013 Action plan for the global strategy for the prevention and control of noncommunicable diseases.

[2] He FJ, Nowson CA, Lucas M, MacGregor GA (2007). Increased consumption of fruit and vegetables is related to a reduced risk of coronary heart disease: meta-analysis of cohort studies Hum Hypertens. Hum Hypertens Meta-Analysis.

[3] Riboli E, Norat T (2003). Epidemiologic evidence of the protective effect of fruit and vegetables on cancer risk. Am J Clin Nutr.

[4] Lock K, Pomerleau J, Causer L, Altmann DR, McKee M (2005). The global burden of disease attributable to low consumption of fruit and vegetables: implications for the global strategy on diet. Bull World Health Organ.

[5] Begg S, Vos T, Barker B, Stevenson C, Stanley L, Lopez A (2007). The burden of disease and injury in Australia 2003.

[6] Rayner M, Scarborough P (2005). The burden of food related ill health in the UK. J Epidemiol Community Health.

[7] National Health and Medical Research Council (2013). Australian Dietary Guidelines.

[8] Hall JN, Moore S, Harper SB, Lynch JW (2009). Global variability in fruit and vegetable consumption. Am J Prev Med.

[9] World Health Organization (2013). WHO global Infobase: fruit and vegetable consumption.

[10] Australian Bureau of Statistics (2012). Australian health survey: first results, 2011–12 Cat no. 4364.0.

[11] Australian Bureau of Statistics (1998). National nutrition survey intake and physical activity measurements Australia 1995. Cat no 4805.0.

[12] Australian Institute of Health and Welfare (2012). Australia's food and nutrition.

[13] Rangan AM, Schindeler S, Hector DJ, Gill TP, Webb KL (2009). Consumption of ‘extra’ foods by Australian adults:types, quantities and contribution to energy and nutrient intakes. Eur J Clin Res.

[14] Australian Bureau of Statistics (2013). Gender indicators, Australia. Canberra, Cat no. 4125.0.

[15] Nuss H, Freeland-Graves J, Clarke K, Klohe-Lehman D, Milani TJ (2007). Greater nutrition knowledge is associated with lower 1-year postpartum weight retention in low-income women. J Am Diet Assoc.

[16] Reyes NR, Klotz AA, Herring SJA (2013). Qualitative study of motivators and barriers to healthy eating in pregnancy for low-income, overweight, African-American mothers. J Acad Nutr Diet.

[17] Eikenberry N, Smith C (2004). Healthful eating: perceptions, motivations, barriers, and promoters in low-income minnesota communities. J Am Diet Assoc.

[18] Chang MW, Nitzke S, Guilford E, Adair CH, Hazard DL (2008). Motivators and barriers to healthful eating and physical activity among low-income overweight and obese mothers. J Am Diet Assoc.

[19] Bastian LA, Pathiraja VC, Krause K, Namenek Brouwer RJ, Swamy GK, Lovelady CA, Ostbye T (2010). Multiparity is associated with high motivation to change diet among overweight and obese postpartum women. Womens Health Issues.

[20] Peterson KE, Sorenson G, Pearson M, Hebert JR, Gottlieb BR, McCormick MC (2001). Design of an intervention addressing multiple levels of influence on dietary and activity patterns of low-income, postpartum women. Health Educ Res.

[21] Baker J (2011). Developing a care pathway for obese women in pregnancy and beyond. Br J Midwifery.

[22] De Jerse SJ, Ross LJ, Himstedt K, McIntyre HD, Callaway LK (2011). Weight gain and nutritional intake in obese pregnant women: some clues for intervention. Nutr Diet.

[23] Keller C, Records K, Ainsworth B, Permana P, Coonrod DV (2008). Interventions for weight management in postpartum women. J Obstet Gynecol Neonatal Nurs.

[24] Ostbye T, Krause KM, Brouwer RJN, Lovelady CA, Morey MC, Bastian LA, Peterson BL, Swamy GK, Chowdhary J, McBride CM (2008). Active mothers postpartum (AMP): rationale, design, and baseline characteristics. J Womens Health.

[25] Jones C, Burns S, Howat P, Jancey J, McManus A, Carter O (2010). Playgroups as a setting for nutrition and physical activity interventions for mothers with young children: exploratory qualitative findings. Health Promot J Aust.

[26] Council NHMRC (2003). Dietary guidelines for Australian adults.

[27] Glanz K, Bishop D (2010). The role of behavioral science theory in development and implementation of public health interventions. Annu Rev Publ Health.

[28] Prochaska J, Diclemente C (1983). Stages and processes of self-change of smoking - toward an integrative model of change. J Consult Clin Psychol.

[29] Rollnick S, Miller WR (1995). What is motivational interviewing?. Behav Cogn Psych.

[30] Jancey J, Howat P, Burns S, Jones C, Dhaliwal S, McManus A, Hills A, Anderson A (2011). The protocol of a randomized controlled trial for playgroup mothers: Reminder on Food, Relaxation, Exercise, and Support for Health (REFRESH) Program. BMC Public Health.

[31] Wright J, Scott J (2000). The fat and fibre barometer, a short food behaviour questionnaire: reliability, relative validity and utility. Aust J Nutr Diet.

[32] Marks G, Webb K, Rutishauser I, Riley M (2001). Monitoring food habits in the Australian population using short questions: Australian Food and Nutrition Monditoring Unit. Ausinfo.

[33] National Cancer Institute (2003). National health and nutrition examination survey (NHANES) NHANES food frequency questionnaire U.S. National Institutes for Health.

[34] Campbell MK, Elbourne DR, Altman DG, CONSORT group (2004). CONSORT statement: extension to cluster randomised trials. BMJ.

[35] DeBoer MD, Scharf RJ, Demmer RT (2013). Sugar-sweetened beverages and weight gain in 2-to 5-year-old children. Pediatrics.

[36] Thompson JL, Allen P, Helitzer DL, Qualls C, Whyte AN, Wolfe VK, Herman CJ (2008). Reducing diabetes risk in American Indian women. Am J Prev Med.

[37] Havas S, Anliker J, Greenberg D, Block G, Block T, Blik C, Langenberg P, DiClemente C (2003). Final results of the Maryland WIC food for life program. Prev Med.

[38] Craigie AM, Macleod M, Barton KL, Treweek S, Anderson AS (2011). Supporting postpartum weight loss in women living in deprived communities: design implications for a randomised control trial. Eur J Clin Nutr.

[39] Fahrenwald NL, Sharma M (2002). Development and expert evaluation of “Moms on the Move”, a physical activity intervention for WIC mothers. Publ Health Nurs.

[40] Fjeldsoe BS, Miller YD, Marshall AL (2010). Mobile Mums: a randomized controlled trial of an SMS-based physical activity intervention. Ann Behav Med.

[41] Lombard CB, Deeks AA, Ball K, Jolley D, Teede HJ (2009). Weight, physical activity and dietary behavior change in young mothers: short term results of the HeLP-her cluster randomized controlled trial. Nutr J.

[42] Miller YD, Trost SG, Brown WJ (2002). Mediators of physical activity behavior change among women with young children. Am J Prev Med.

[43] O'Toole ML, Sawicki MA, Artal R (2003). Structured diet and physical activity prevent postpartum weight retention. J Womens Health.

[44] Cena ER, Joy AB, Heneman K, Espinosa-Hall G, Garcia L, Schneider C, Wooten Swanson P, Hudes M, Zidenberg-Cherr S (2008). Learner-centered nutrition education improves folate intake and food-related behaviors in nonpregnant, low-income women of childbearing age. J Am Diet Assoc.

[45] Cramp AG, Brawley LR (2006). Moms in motion: a group-mediated cognitive-behavioral physical activity intervention. Int J Behav Nutr Phys Act.

[46] Gaston A, Prapavessis H (2009). Maternal-fetal disease information as a source of exercise motivation during pregnancy. Health Psychol.

[47] Hausenblas HA, Brewer BW, Van Raalte JL, Cook B, Downs DS, Weis CA, Cruz A (2008). Development and evaluation of a multimedia CD-ROM for exercise during pregnancy and postpartum. Patient Educ Counsel.

[48] Polley BA, Wing R, Sims C (2002). Randomized controlled trial to prevent excessive weight gain in pregnant women. Int J Obes Relat Metab Disord.

[49] Huang TT, Yeh CY, Tsai YC (2011). A diet and physical activity intervention for preventing weight retention among Taiwanese childbearing women: a randomised controlled trial. Midwifery.

[50] Krummel D, Semmens E, MacBride AM, Fisher B (2010). Lessons learned from the mothers' overweight management study in 4 West Virginia WIC offices. J Nutr Educ Behav.

